# Metabolism-Associated Epigenetic and Immunoepigenetic Reprogramming in Liver Cancer

**DOI:** 10.3390/cancers13205250

**Published:** 2021-10-19

**Authors:** Chaofan Fan, Shing Kam, Pierluigi Ramadori

**Affiliations:** Division of Chronic Inflammation and Cancer, German Cancer Research Center (DKFZ), 69120 Heidelberg, Germany; shing.kam@dkfz-heidelberg.de

**Keywords:** liver cancer, immunometabolism, Warburg effect, acetyl-CoA, SAM, resminostat, metformin, guadecitabine, combinatorial therapy

## Abstract

**Simple Summary:**

Liver cancer is one of the most frequently occurring cancer types and one of the leading causes of cancer-related mortality globally. Despite of its constantly growing incidence, the efficacy of the current therapeutic interventions is limited. Metabolic and epigenetic aberrations are two distinct but mutually influential factors which contribute to the plasticity and adaptability of tumor cells to a hostile microenvironment, leading to high resilience and permissive conditions. This review offers the most up-to-date overview of the recently reported major metabolic dysregulations in liver cancer cells, the consequential epigenetic reprogramming, as well as the opportunity to explore the effect on metabolic competition and inhibition of immune cells in the tumor microenvironment. Finally, we discuss the potential therapeutic value of pharmaceutical inhibition of these essential pathways in combating liver cancer.

**Abstract:**

Metabolic reprogramming and epigenetic changes have been characterized as hallmarks of liver cancer. Independently of etiology, oncogenic pathways as well as the availability of different energetic substrates critically influence cellular metabolism, and the resulting perturbations often cause aberrant epigenetic alterations, not only in cancer cells but also in the hepatic tumor microenvironment. Metabolic intermediates serve as crucial substrates for various epigenetic modulations, from post-translational modification of histones to DNA methylation. In turn, epigenetic changes can alter the expression of metabolic genes supporting on the one hand, the increased energetic demand of cancer cells and, on the other hand, influence the activity of tumor-associated immune cell populations. In this review, we will illustrate the most recent findings about metabolic reprogramming in liver cancer. We will focus on the metabolic changes characterizing the tumor microenvironment and on how these alterations impact on epigenetic mechanisms involved in the malignant progression. Furthermore, we will report our current knowledge about the influence of cancer-specific metabolites on epigenetic reprogramming of immune cells and we will highlight how this favors a tumor-permissive immune environment. Finally, we will review the current strategies to target metabolic and epigenetic pathways and their therapeutic potential in liver cancer, alone or in combinatorial approaches.

## 1. Introduction

Liver cancer is the fourth most common cause of cancer death worldwide [1]. No effective liver cancer therapy has been approved so far and therefore there is an urgent need to find efficient and safe therapeutic strategies. Liver cancer is induced by several pathological processes normally related to chronic inflammation, such as metabolic and nutritional disturbances (ASH/NASH) as well as viral infections (HBV/HCV) [2]. The central feature of chronic liver disease, defined as necro-inflammation, is characterized by a persistent inflammatory process originating in response to hepatic cell death [3]. A chronic insult generating a compensatory loop of cell proliferation within an altered immune-microenvironment triggers irreversible DNA damage to promote cellular transformation. In this context, the adaptive metabolic changes occurring in hepatocytes are not only essential in sustaining the cellular transformation but also determine the development of a tumor-permissive microenvironment.

Except rare forms of hepatoblastoma, liver cancer can be substantially categorized in two entities: hepatocellular carcinoma (HCC) and cholangiocellular carcinoma (CCA). These two types of cancer are not only characterized by a substantial difference in the cell type acquiring malignant character, but they also display diverse mutational landscapes associated with defined metabolic phenotypes shaping different tumor microenvironments [4].

Over the years, the most challenging aspect in the identification of druggable therapeutic targets in liver cancer turned out to rely on the heterogeneity of intrinsic properties of cancer cells (e.g., genomic mutations) and of extrinsic factors in the microenvironment affecting tumor development and progression. This concept, that is not only tissue-specific but also closely related to the nature of the oncogenic insult, can be extended to a temporal dimension encompassing the different stages of cancer development. In fact, through an enduring dynamic process, each stage of the disease can be characterized by different genetic (epigenetic), metabolic, and immune profiles.

Generally, conditions leading to hepatocyte stress or damage inevitably result in alterations of its metabolic activity. Vice-versa, loss of the energetic balance could impair the functionality of cellular organelles leading to hepatocyte damage. This bidirectional process becomes clear in the context of chronic liver injury, where the constant cell damage alternates to a compensative regenerative stimulus. Consequently, the cellular metabolism has to cope with the energetic demand of stressed and proliferative hepatocytes [5]. Accordingly, the oncogenic transformation of hepatocytes towards a malignant phenotype is characterized by dramatic changes of the cellular metabolism referred to as metabolic reprogramming. This capability of transforming cells to adapt their metabolic activity to the altered proliferative and growth rate as well as to challenging microenvironments has been identified as a hallmark of cancer and presents cancer-specific profiles. It is nowadays clear from experimental models and clinical evidence that many oncogenic mutations selectively induce metabolic changes that critically sustain tumor growth. A list of the most frequent mutations influencing cellular metabolism is here reported in Table 1. However, in liver cancer, not only the genomic landscape but also the immune characteristics of transforming cells frequently define the microenvironment that dictates a distinct metabolic profile of the tumor as, for instance, in the case of NASH-related HCC [6]. In turn, metabolites produced by cancer cells generate a permissive immune-microenvironment for favorable growing conditions. Thus, most of the metabolic pathways regulating the energetic balance undergo changes conferring particular resistance against hostile growth conditions and generating an immunosuppressive terrain.

Per definition, epigenetic regulation of a gene is an inherited character that affects a phenotype by interfering with the gene expression without altering the DNA sequence [18,19]. As an essential part of the evolutionary process, it mirrors the capacity of adaptation to the microenvironment and the versatility of the cell to respond to extracellular changes. The epigenetic machinery substantially comprehends several families of enzymes that shape the chromatin conformation and the DNA-histones interaction enabling or hindering the access of transcription factors to the genome. Alterations of the cellular metabolism occurring in transforming malignant cells markedly influence the epigenetic mechanisms and, in return, epigenetic modifications commonly sustain the metabolic reprogramming through the regulation of specific genes [20].

In this review, we will describe the most important changes of the metabolism characterizing and supporting hepatocarcinogenesis. We will illustrate how these changes of cellular metabolism impact on epigenetic modifications highlighting the most recent findings about the major alterations of the chromatin occurring in liver cancer cells. Successively, we will describe how metabolic reprogramming of liver cancer can possibly influence the tumor immune-microenvironment and how epigenetic alterations of the immune cells can affect cancer growth. Finally, we will review the most novel findings about current therapeutic strategies aiming to modulate cancer-specific metabolic pathways and epigenetic modifications in order to render cancer more vulnerable to conventional therapies.

## 2. Metabolic Reprogramming in Liver Cancer

**Figure 1:**
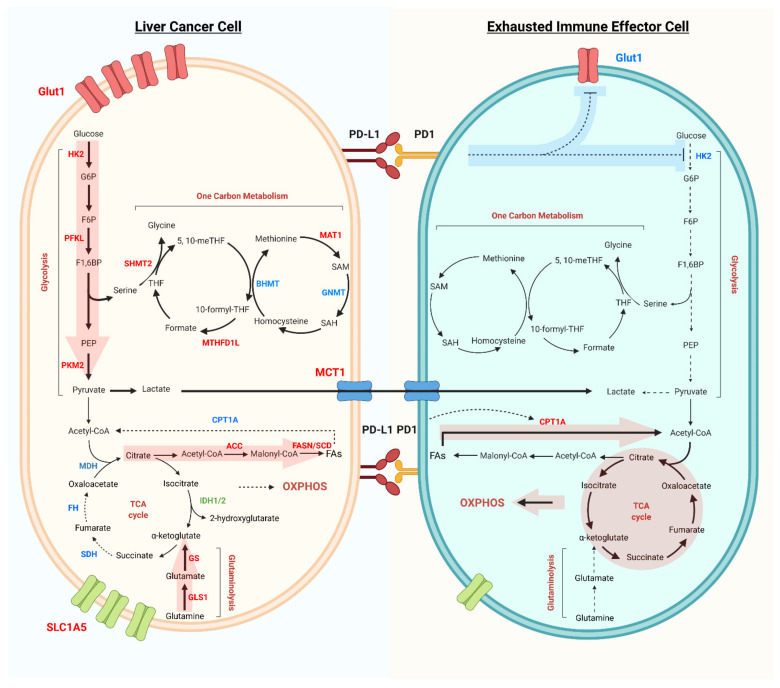
Metabolic reprogramming of liver cancer cells and exhausted immune effector cells in the tumor microenvironment. Liver cancer cells have increased glycolysis flux, as well as increased expression of Glut1, rate-limiting enzymes HK2, PFKL, and PKM2 (red). Glutaminolysis and fatty acids synthesis are the other two main pathways important for tumor cell survival and proliferation. While the TCA cycle and fatty acid oxidation, as well as the rate-limiting enzymes like SDH, FH, MDH, and CPT1A (blue) are downregulated in liver cancer cells. Metabolites, such as lactate, can be exported and influence the tumor infiltrated immune cells. In addition, tumor cell expressing PD-L1 can activate PD-1 on immune cells, inhibiting glucose uptake (Glut1) and Glycolysis (HK2). Dysregulated glycolysis, increased fatty acid oxidation and OXPHOS are the main metabolic phenotypes of exhausted immune effector cells.

Metabolic reprogramming in cancer cells is therefore driven by a necessity to compensate energy expenditures related to increased cell proliferation and cell division. This is functionally translated in boosting pathways that result in efficient and fast energy production such as glycolysis and TCA cycle as well as reactions that easily generate essential cellular components, like amino acid and one-carbon metabolism. These finely interconnected pathways lead on one hand to a condition of extra-cellular nutrients deprivation and on the other hand to accumulation of specific metabolites (e.g., lactate). In the surroundings, differentiation, polarization, and activation of immune cells, which frequently correlate with increased glycolytic fluxes and augmented oxidative phosphorylation, strongly depend on the availability of substrates catalyzing these metabolic reactions (Figure 1). The impact of the metabolic microenvironment on the progression from chronic liver disease to cancer was recently reviewed elsewhere [21]. However, here we shortly summarize the metabolic pathways majorly affected by reprogramming and directly implicated in the epigenetic changes described in the next sections.

### 2.1. Glycolysis

Anaerobic glycolysis is the first form of metabolic reprogramming described in liver cancer. In fact, this is the quickest way to provide ATP via glucose consumption in unfavorable conditions of growth at the expenses of a reduced mitochondrial oxidative phosphorylation. These conditions are typically characterized by decreased O_2_ availability due to scarce intratumoral vascularization and increased substrates consumption from hyper-proliferating cancer cells [21]. Thus hypoxia is a central determinant of changes affecting glucose metabolism, triggering a switch from oxidative to glycolytic metabolism [22]. In fact, the expression of the most important rate limiting enzymes involved in glycolysis is frequently altered in liver cancer [23]. Most of these genes are regulated by the O_2_-sensitive transcription factor hypoxia inducible factor 1 (HIF1α) that was reported to be stabilized and activated in HCCs [24]. Of note, HIF1α also regulates the expression of the glucose transporter GLUT1 which resulted to be upregulated in HCC and to be associated with tumor histological grade [25]. The first glycolytic enzyme that converts glucose into glucose-6-phosphate (G-6-P) is named hexokinase (HK). The expression of the isoform 2 (HK2) was reported to be elevated in human HCCs and its genetic ablation in murine models of hepatocarcinogenesis was shown to decrease tumor incidence and cancer cell proliferation [26]. Of note, inhibition of glycolysis, as a consequence of HK2 inhibition, resulted in re-activation of oxidative phosphorylation. Another rate-limiting enzyme of the glycolytic pathway, phosphofructokinase (PFKL), was recently found to be over-expressed in tumor tissues as compared to peri-tumoral tissue in liver biopsies of HCC-diagnosed patients [27]. Similarly, downstream of the glycolytic pathway, the pyruvate kinase PKM2 was shown to be highly upregulated in the liver of HCC patients where it was associated with poor prognosis [28], and also overexpressed in CCA tissues and cell lines [29]. In summary, the importance of the metabolic switch favoring glycolysis was recently highlighted in a functional study showing that over-expression of the RNA-helicase MTR4 is required to induce tumorigenesis in the context of HCC and this depends on the regulation of a metabolic reprogramming sustaining the glycolytic pathway [30].

### 2.2. TCA Cycle and Lipid Metabolism

The tricarboxylic acid cycle (TCA) is a central metabolic pathway regulating cellular energy production and contributing to the synthesis of essential macro-molecules as well as to the maintenance of the cellular redox balance. The cycle utilizes acetyl-CoA derived from the dehydrogenation of pyruvate to convert it to citrate that is transformed in the final product oxaloacetate (OAA) with the generation of NADH and CO_2_. The reduced NADH fuels the oxidative phosphorylation to produce ATP and many intermediate metabolites that are substrates for other metabolic pathways. For these reasons, the TCA cycle activity and the expression of several rate-limiting enzymes were shown to be altered in the liver of patients with HCC [31]. α-ketoglutarate (α-KG) also originates in the TCA cycle from isocitrate. This reaction is catalyzed by the cytoplasmic enzyme isocitrate dehydrogenase 1 (IDH1) with reduction of NADP into NADPH. Gain of function mutations of the isoforms IDH1 and IDH2 are frequently observed in human CCA [32]. Downregulation of the succinate dehydrogenase (SDH) was often reported in HCCs and this is related to increased levels of succinate in patients with HCC [33]. Similarly, fumarate hydratase (FH), enzyme that facilitates the conversion of fumarate to OAA, was also reported to be downregulated in a small cohort of HCC patients [34]. Of interest, mutations of FH as well as SDH might be associated with epigenetic alterations characterized by DNA methylation of specific regions coding for tumor suppressor genes [35]. Downregulation of malate dehydrogenase (MDH), the enzyme catalyzing the final step of the TCA cycle to re-generate OAA from malate, was also detected in the early phases of HBx-driven hepatocarcinogenesis in mice [36].

As an important source of energetic substrates, abnormalities of lipid metabolism have been abundantly reported in the context of liver cancer. In particular, de novo lipogenesis was shown to be preferentially used as a source of fatty acids by hepatic cancer cells [37]. For instance, an integrative proteomic/transcriptomic analysis demonstrated that HCC display a quite heterogeneous metabolic profile [38]. The authors showed specific increase of de novo lipogenesis in most of the samples in a cohort of 356 HCC tumors compared to adjacent non-cancerous tissue. Interestingly, they individuated mitochondrial acetate as a major substrate utilized for lipid synthesis and a significant upregulation of the mitochondrial enzyme acetyl-CoA Sythetase-1 (ACSS1) in HCC. In this regard, acetate is a critical substrate for reactions involved in epigenetic modifications as illustrated later in this review. Similarly, genetic deletion of fatty acids synthesis (FASN) was shown to inhibit hepatocarcinogenesis in murine models and proliferation in hepatoma cell lines [39]. However, the beneficial effects of lipogenic inhibition on cancer growth seem to be dependent on the oncogenes driving the process as well as on the microenvironmental profile [40]. In line with the concept that fatty acids catabolism constitutes an important source of energy for proliferating cells, some HCC also display increased rates of fatty acids oxidation (FAO). CTNNB1-mutated HCC seems to promote FAO rather than glycolysis and this phenotype is dependent on PPAR-α expression as shown in transgenic mouse models and CTNNB1-mutated human HCCs [41]. Accordingly, it was shown that activation of the transcription factor NANOG triggers a switch from oxidative phosphorylation to FAO through a metabolic reprogramming critical to support the growth of tumor-initiating stem-like cells in NASH/ASH-induced and HCV-related HCC [42]. Moreover, in a fat-enriched microenvironment as in NAFLD context, cancer cells develop a particular resistance to anti-angiogenic therapy through induction of FAO and fatty acids uptake, enabling them to survive hypoxic environments [43].

### 2.3. Amino Acids Metabolism

Beyond lipid and glucose metabolism, another relevant source of carbon in highly proliferating cells derives from the amino acids metabolism [44]. In fact, amino acids are not only addressed to protein synthesis or nucleic acids synthesis but they can also represent an alternative source of energy through the production of α-ketoacid that is oxidized in the TCA cycle and they can participate in the maintenance of the cellular redox balance (e.g., glutathione synthesis). Glutamine, that can be synthetized directly from glucose, is the most abundant amino acid consumed by liver cancer cells at high rates [45]. Glutamine synthetase (GS) is the enzyme responsible for glutamate conversion to glutamine and it is frequently overexpressed in liver cancer associated with CTNNB1 mutations [19]. On the other hand, glutamine is catabolized by the enzyme glutaminase (GLS) into glutamate that is dehydrogenated into α-KG to fuel the TCA cycle. Whereas the isoform GLS1 was reported to be overexpressed in intra-hepatic CCA [46], the expression of GLS2 was shown to inversely correlate with tumor size and prognosis in human HCC [47]. Glutamate is also a precursor of glutathione (GSH) that is a key molecule in the control of redox homeostasis. In this regard, it was shown that myc-driven liver tumors are particularly sensitive to oxidative stress because of their reduced glutathione production [48].

Experimental models of liver carcinogenesis recently indicated that also the metabolism of the non-essential amino acid proline seems to be altered in HCC and the expression of the enzyme pyrroline-5-carboxylate reductase 1 (PYCR1) involved in its biosynthesis is also increased in human HCC and directly correlated with tumor grade [49].

As learned by experimental murine models of cancer, deficiency of S-adenosyl-L-methionine (SAM) resulting in DNA hypomethylation is capable to induce liver cancer in animals fed a methionine-deficient diet. Similarly, human HCC and liver tumors from transgenic c-Myc were shown to display a defective methionine metabolism with increased level of the enzyme S-adenosylmethionine synthase isoform type-1 (MAT-1) [50].

Overall, the analysis of serum metabolites of patients diagnosed with hepatobiliary cancer revealed an inverse association with cancer risk for circulating leucine, lysine, and glutamine, whereas a direct association for phenylalanine, tyrosine, and glutamate [51].

### 2.4. One-Carbon Metabolism

In relation to cancer-related alterations of amino acid metabolism, one-carbon metabolism also represents an important pathway for nucleotide synthesis and reducing agents generation as well as a substrate donor for methylation reactions. At the crossroad between folate metabolism and methionine cycle, this pathway results in generation of purine and pyrimidine nucleotides, supports SAM production via serine catabolism and provides NADPH as substrate for redox reactions through the generation of tetrahydrofolate (THF). Many of the enzymes regulating the one-carbon metabolism were reported to be altered not only in HCC but also in the context of chronic liver diseases, such as NASH and ASH [52]. The enzyme methylenetetrahydrofolate dehydrogenase 1–like (MTHFD1L), critically involved in the folate cycle and responsible for the redox transformation of THF, was recently shown to play an essential role in hepatoma cell lines growth and proliferation as well as to be overexpressed in the liver of HCC patients [53]. Similarly, overexpression of the mitochondrial isoform MTHFD2 was reported to be associated with poor prognosis and increased tumor invasion in patients with HCC [54]. On the same line, increased expression of serine hydroxymethyltransferase 2 (SHMT2), enzyme catalyzing the conversion of serine to glycine, was detected in HCC patients and was shown to promote proliferation in HepG2 hepatoma cell lines [55]. Interestingly, high levels of SHMT2 were also reported to correlate with poorer survival in patients with CCA [56]. Furthermore, it is important to note that hypoxia in liver cancer might also have a strong impact in the regulation of many mitochondrial enzymes involved in one-carbon metabolism [57], therefore, contributing to sustain cell growth and survival. Finally, beyond the above mentioned alteration of MAT-1, also the expression of other two important enzymes involved in the methionine cycle, betaine homocysteine methyltransferase (BHMT) and glycine methyltransferase (GNMT) was reported to be downregulated in human and experimental models of HCC [58].

## 3. Metabolic Reprogramming Leading to Epigenetic Changes in Tumor Cells

**Figure 2:**
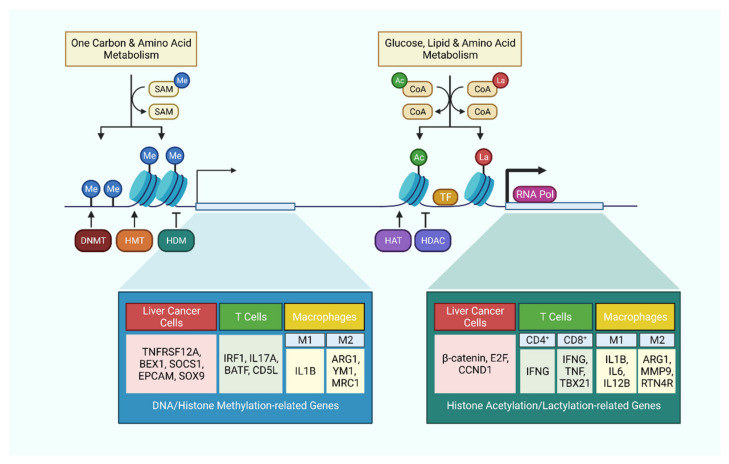
Metabolism-related epigenetic modifications in liver cancer cells and immune cells. Cellular metabolism tightly regulates epigenetic modifications by supplying essential substrates and co-factors. These epigenetic modifications in turn regulate the expression level of critical genes, which not only affect the behavior of cancer cells, but also dictates the activation or polarization of immune cells.

Metabolic reprogramming accompanying hepatic carcinogenesis not only affects cell functionality and activity by changing the energetic balance and the availability of substrates, but it also influences the expression of genes regulating cell metabolism. This transcriptional tuning occurs mainly through the accumulation of metabolites that boost the enzymatic activity of epigenetic regulators. The most common metabolites that function as substrate for chromatin modifications are SAM, acetyl-CoA and nicotinamide adenine dinucleotide (NAD^+^) as well as α-KG, and are tightly dependent on alterations of the metabolic pathways previously described. They are responsible for most of the acetylation and methylation processes of DNA and histones and they are commonly altered in the context of hepatocarcinogenesis (Figure 2).

### 3.1. DNA Methylation

As in many other cancer types, abnormal hypermethylation and hypomethylation of chromatin components, DNA and histones, are frequent alterations occurring in liver cancer [18,20]. SAM functions as a methyl-donor and therefore as a substrate for the family of enzymes named as DNA methyltransferases (DNMTs). Gene expression of the isoforms DNMT1, DNMT3a, and DNMT3b was shown to be upregulated in correlation with the number of methylated genes in HCCs as compared to normal liver [59]. Emerging evidence indicates that alcohol can induce hepatocarcinogenesis also through epigenetic changes, in particular related to alteration of DNA methylation [60]. It was recently shown that the DNA methyltransferase DNMT3L, might be responsible for the methylation of TNFRSF12A, which is overexpressed and methylated in alcohol-related HCC patients [61]. Moreover, a recent study showed that DNMT1 regulates the expression of BEX1, a gene necessary for the maintenance of tumor stemness and resistance to chemotherapy in HCC [62] (Figure 2). On the other hand, the glycine-N-methyltransferase (GNMT) was reported to be downregulated in HCC and its genetic deficiency was shown to trigger DNA hypermethylation, liver fibrosis, and cancer in mice [63]. Demethylation of DNA is also catalyzed by the ten-eleven translocation family proteins (TETs). Recent findings indicate that TET downregulation might be involved in the promotion of metastasis in HCC via demethylation inhibition of SOCS1 [64]. Interestingly, TET1 protein expression was recently found to be elevated in patients with CCA where it seems to promote malignancy in an α-KG dependent manner [65].

### 3.2. Histone Modifications

The reactions of acetylation that interest mainly histones are catalyzed by histoneacetyltransferases (HATs) that use as a substrate Acetyl-Co-A to acetylate lysine residues present on the protein. A very recent proteomic study showed that HAT1 is significantly elevated in human HCC and CCA [66]. Notably, the authors demonstrated that it can promote cancer growth through succinylation of histones (H3) and non-histone (PGAM1) proteins. On the contrary, histone deacetylases (HDACs) remove the acetyl groups on the lysine residues. The isoform HDAC8 was shown to drive NASH-induced HCC through a de-repression of the β-catenin pathway [67] (Figure 2). Whereas all the several classes of this enzyme were shown to be upregulated in hepatoma cell lines, the expression in human HCC tissues as compared to non-tumoral tissues was indeed differently deregulated [68]. HDAC5 was shown to promote aggressiveness and invasion in HCC whereas increased nuclear frequency of HDAC6 was correlated with poor prognosis in HCC patients [69]. Histones can also undergo methylation modifications that are usually driven by histone methyltransferases (HMTs). Upregulation of the histone methyltransferase G9a was found in human HCC significantly associated with tumor progression and aggressive clinical features [70]. In contrast, histone demethylases (HDM) remove methyl groups from histones. The histone demethylase 4D was reported to be overexpressed in liver cancer and to enhance self-renewal of liver cancer stem-like cells [71].

### 3.3. ATP-Dependent Chromatin Remodeling

A further level of epigenetic regulation is also exerted by ATP-dependent remodeling complexes that enable dynamic restructuring of chromatin density in the nucleosome with energy derived from ATP hydrolysis. ARID2 is one of those complexes that was shown to be negatively correlated with HCC metastasis and positively with patient survival [72]. Mechanistically, ARID2 was shown to recruit DNMT1 to the promoter of Snail1 suppressing the transcription of the gene. A more controversial role was proposed for BRG1 (or SMARCA4), another regulator of the chromatin structure, which was reportedly described to be overexpressed in HCC but with ambiguous functions depending on the oncogenic stimulus [73]. Of the same family, the enzyme helicase lymphoid-specific (HELLS) was also found to be overexpressed in HCC patients and significantly correlated with poor prognosis due to augmented tumor aggressiveness [74]. Of note, experimental knockout of HELLS resulted in a global DNA hypomethylation on the promoter regions of different genes.

## 4. Metabolism of the Immune Cells in the Microenvironment of Liver Cancer

The discussed tumor metabolic reprogramming gives rise to a suboptimal metabolic microenvironment for infiltrated immune cells. These conditions have a pivotal role in driving the exhaustion of immune effector cells, in particular CD8^+^ T cell and NK cell exhaustion, which in turn provokes immune evasion (Figure 1). In contrast, these adverse conditions often favor the activity of anti-inflammatory and tumor-associated immune cells, such as Treg cells, M2 macrophages, and myeloid-derived suppressor cells, all of which might confer further therapeutic resistance. In this section, we will summarize the dysregulation of major epigenetic-related metabolic pathways of tumor infiltrating immune cells and the resulting effects.

### 4.1. Glycolysis

Glycolysis serves as a metabolic hub to interconnect various anabolic pathways, including the pentose phosphate pathway (PPP), hexosamine biosynthetic pathway, fatty acid synthesis, and de novo serine biosynthesis. Glycolytic intermediates divert into these ancillary metabolic pathways to support the production of nucleotides, lipids, and amino acids, supplying adequate biomaterials for cytokine synthesis and sustaining rapid clonal expansion of immune cells. Liver cancer, which is characterized by strong Warburg effect, tends to create a tumor microenvironment (TME) that is devoid of glucose, and hence impairing local immune surveillance through nutritional competition [75].

T cell receptor (TCR) dependent CD8^+^ T cells activation involves an initial switch from oxidative phosphorylation in the resting state, to glycolysis by PDHK1 in early activation, which inhibits mitochondrial import of pyruvate and liberates cytokine mRNA from the inhibitory binding to LDH [76]. This short-term metabolic reprogramming boosts the early translation of IFN-γ, TNF-α, and IL-2 transcripts. Thereafter, long-term glycolytic reprogramming is induced by co-stimulatory signaling of CD28, via the Akt/mTOR and HIF-1α pathway to promote GLUT1-dependent glucose uptake as well as the expression of glycolytic enzymes, which sustains the increasing energetic and biosynthetic demands. Due to the biological importance of the glycolytic pathway, scarcity of glucose in the TME imposes a major hurdle for effector T cell activity. Sole restriction of glucose availability limits CD8^+^ T cell responsiveness, despite the presence of tumor antigenic stimulation [77]. On top of nutrient competition, the regulation by immune checkpoints also generates another level of metabolic disadvantage in T cells (Figure 1). PD-L1 alone already augments glucose metabolism in tumor cells through the upregulation of the Akt/mTOR pathway, depleting the T cells in the TME of glucose [77]. In addition, inhibitory signaling from PD-1 and CTLA-4 further suppresses glycolysis by dampening the PI3K/Akt- and MEK/ERK-mediated expression of both GLUT1 and HK2. Such blockade of glucose intake and utilization results in T cell exhaustion and tolerance [78,79]. Simultaneously, accelerated aerobic glycolysis leads to the export and accumulation of its end product, lactate, into the TME. Lactate dehydrogenases (LDHs) catalyzes the crucial conversion of pyruvate to lactate, and its upregulation is associated with unfavorable prognosis in multiple cancer types, including HCC [80]. However, tumor cells are well adapted to utilize lactate as an alternative energy source, but the immune compartment can be challenged by acidification of the TME [81]. The buildup of lactic acid diminishes the level of the transcriptional activator NFAT, resulting in the impaired IFN-γ secretion in T cells and NK cells, and hence permits immune evasion [82]. Furthermore, hepatic tissue-resident NK cells also appear to be highly sensitive to lactic acid, which prompts mitochondrial dysfunction, and ultimately, apoptosis [83,84]. HCC glycolysis therefore interferes with the function of the innate immune system in the TME, ablating interferon signaling and NK cell cytotoxicity [85].

Immune checkpoint blockade normalizes the altered glucose metabolism and invigorates exhausted T cells, which might be partly attributed to the activation of PPARγ target genes [77,79]. On the other hand, direct inhibition of tumor glycolysis can further sensitize tumor cells to immune checkpoint therapy [86]. Interestingly, recent studies suggested that manipulation of other related metabolic pathways could also indirectly enhance the glycolytic phenotype of immune cells and retain their fitness in the TME. Autophagy was reported to be antagonistic to cytokine production and glucose uptake in CD8^+^ T cells, whereas the abrogation of it promoted glucose uptake as well as tumoricidal activity [87]. Blockade of the lower oxidative PPP in T cells also enhanced tumor clearance, via mROS-induced activation of the Nrf2 pathway [88]. In coherence, Nrf2 activation was also demonstrated to support the metabolic flexibility of NK cells and maintain their glycolytic rate in the hostile TME [89].

### 4.2. TCA Cycle, Glutaminolysis, and Fatty Acid Oxidation

As discussed, deprivation of glucose in the TME and immune checkpoint signaling can both suppress glycolysis, forcing immune cells to switch to fatty acid oxidation (FAO) and oxidative phosphorylation (OXPHOS). The overexpression of the FAO rate-limiting enzyme CPT1A by PD-1 signaling has been shown to be responsible for such a shift in energy production (Figure 1). OXPHOS, however, is a major source of intracellular ROS, and insufficient clearance can lead to oxidative stress. Repetitive antigenic stimulation triggers defects in mitochondrial biogenesis and mitophagy. Loss of mitochondrial function results in the subsequent elevation of intra-cellular ROS level, which prevents the dephosphorylation of NFAT, causing the persistent signaling that eventually drives T cell exhaustion [90,91,92]. Conversely, regulatory T cells (Treg) appear to be able to thrive in the TME, despite of the adverse nutritional context. Treg Foxp3 represses Myc transcription to downregulate glycolysis and upregulate OXPHOS. This metabolic reprogramming not only reduces the dependence on glucose intake, but also contributes to a higher NAD^+^: NADH ratio, enabling the oxidation of exogenous lactate into pyruvate for driving the TCA cycle [93].

Apart from channeling NADH and FADH_2_ to the electron transfer chain for energy production, the TCA cycle also generates essential intermediate metabolites that play crucial biological functions in immune cells. The polarization of macrophages, in particular, depends highly on the balance of TCA cycle intermediates. α-KG has been reported to confer an anti-inflammatory M2 phenotype, possibly by restraining NF-κB and destabilizing HIF-1α, whereas succinate is linked to the pro-inflammatory M1 phenotype [94]. Glutamine is one of the major carbon sources in cells, and it fuels the TCA cycle in the form of α-KG generated from glutaminolysis. Abolishment of this catabolic process skews macrophage polarization towards the M1 phenotype, and leads to the reduction of myeloid-derived suppressor cells (MDSCs), rendering the tumor more susceptible to immune checkpoint therapy [94,95]. Itaconate is another anti-inflammatory metabolite which is closely tied to the TCA cycle. IRG1 catalyzes the production of itaconate from aconatate. Itaconate hampers OXPHOS by inhibiting SDH, while it supports the activation of Nrf2 and contributes to innate immune tolerance, which is a mechanism that seems to be preserved also in Kupffer cells [96,97,98].

### 4.3. One-Carbon Metabolism

Methionine influx and metabolism are crucial for T cell activation upon stimulation of antigen receptors, nucleic acids, and reducing agents, together with crucial precursors and co-factors to maintain critical biological processes [99]. As described above, the one-carbon metabolism has been previously reported to be upregulated in malignant cells, including liver cancer. New findings suggested that not only can such tumoral metabolic alteration grant intrinsic advantage, it can indeed create an immunosuppressive TME to drive cancer progression. A recent study suggested that the increase in folate cycle activity via MTHFD2 also supplies UDP-GlcNAc to enable O-GlcNAcylation-mediated c-MYC stabilization, and thus promote PD-L1 transcription to limit CD8^+^ T cell cytotoxicity in HCC [100]. Besides, HCC cells with upregulated one-carbon metabolism release intermediate metabolites into the TME, namely 5-methylthioadenosine (MTA) and S-adenosylmethionine (SAM), which in turn precipitate T cell exhaustion [101]. The serum levels of these metabolites therefore also appear to serve as prognostic indicators of the HCC metabolic reprogramming and predict HCC progression [101]. Furthermore, the one-carbon metabolism produces glutathione (GSH), and also NADPH which maintains the reduced state of GSH. Notably, GSH is required to mitigate the ROS triggered by TCR-mediated T cell activation. This antioxidant mechanism upregulates MYC production via the NFAT-mTOR axis to initiate reprogramming of glucose metabolism and facilitate T cell proliferation and cytokine secretion [102].

## 5. Metabolism-Regulated Immunoepigenetic Changes in Liver Cancer

Similar to the case of cancer cells, the aforementioned metabolic aberrations also contribute to the epigenetic reprogramming of immune cells. Notably, these heritable and long-lasting changes can cause a chronic exhaustion phenotype in effector cells, which might not simply be reverted by immune checkpoint blockade alone, leading to the development of nonresponsiveness [103,104]. Here, we highlight the epigenetic changes of immune cells under the context of tumor-induced metabolic reprogramming.

### 5.1. Glycolysis and TCA Cycle-Associated Epigenetic Modifications

Acetyl-CoA is a metabolic intermediate connecting glycolysis to the TCA cycle. It can be produced from the oxidation of pyruvate by pyruvate dehydrogenase, the conversion of citrate by ATP citrate lyase (ACLY), or β-oxidation. In addition to its metabolic function, acetyl-CoA is also the substrate for histone acetylation. TLR activation in macrophages induces upregulation of glycolysis and ATP citrate lyase activity to yield acetyl-CoA, which facilitates the histone H3 and H4 acetylation of specific innate immune responsive gene sets to increase enhancer accessibility [105]. A similar mechanism was also observed in CD4^+^ T cells, where activated T cells upregulate LDHA expression to maximize aerobic glycolysis. This results in histone H3 acetylation of both the promoter and enhancer of IFN-γ, and ultimately promotes effector T cell differentiation [106]. Nonetheless, the production of acetyl-CoA is not limited to self-generating mechanisms. Under glucose deprivation, CD8^+^ T cells were shown to uptake extracellular acetate through MCT-1 and MCT4, and then converted it into acetyl-CoA by ACSS2 for histone H3 and H4 acetylation of cytokine genes to maintain effector function in adverse conditions [107] (Figure 2).

α-KG functions as a co-factor for DNA and histone demethylases, and thus its availability often determines the DNA and histone methylation landscape. α-KG production in HCC was described to favor the development of immune escape, through upregulating the expression of PD-L1 by enhancing TET1-dependent DNA demethylation of the IRF1 transcription activator promoter region [108]. JMJD3, the demethylase which dictates the M2 phenotype of macrophages, also relies on the supply of α-KG [94]. Intriguingly, mutations of the catalytic enzymes IDH1/2 define a subset of CCA, which generates the oncometabolite 2-hydroxyglutarate instead of α-KG, and the associated hypermethylation has been investigated [32]. The 2-hydroxyglutarate in the TME also seems to exert profound effects in shaping the immune landscape, but its relevance in the liver TME remains to be explored [109,110].

Recently a novel histone modification, lactylation, has been first identified [111]. This refers to the attachment of a lactyl group to the lysine residue of histones. Histone lactylation positively correlates with glycolytic rate, and displays a distinct temporal dynamics as compared to histone acetylation. Intriguingly, histone lactylation induces a late-phase M2 phenotype in macrophages, plausibly by stimulating gene transcription in a p53 and p300-dependent fashion.

### 5.2. One-Carbon Metabolism-Associated Epigenetic Modifications

Mechanistically, the one-carbon metabolism-related fate determination of immune cells is not only closely linked to metabolic reprogramming but also the resulting epigenetic rearrangement. The metabolite intermediate of the methionine cycle, SAM, plays a major role as the universal methyl donor in DNA and histone methylation. As such aberration in the one-carbon metabolism can be associated with hypomethylation of important genes. For instance, in HCC, high concentration of SAM and MTA leads to lowered accessibility of key T cell activation gene sets which are involved in lymphocyte proliferation and differentiation, and hence skewing towards an exhaustion phenotype [101]. In T helper cells, SAM is mainly synthesized from extracellular methionine, and its level determines the global H3K4me3 histone methylation, and the expression of lineage-specific genes that drives TH17 cell differentiation, expansion, and pro-inflammatory function [112]. Activated macrophages fuels the folate cycle and methionine cycle by corporately upregulating glycolysis and de novo serine biosynthesis. The production of SAM induces H3K4me3 and H3K36me3 histone methylation to enhance occupancy of IL-1β and other pro-inflammatory genes [113] (Figure 2).

## 6. Metabolic Targets in the Treatment of Liver Cancer

**Figure 3:**
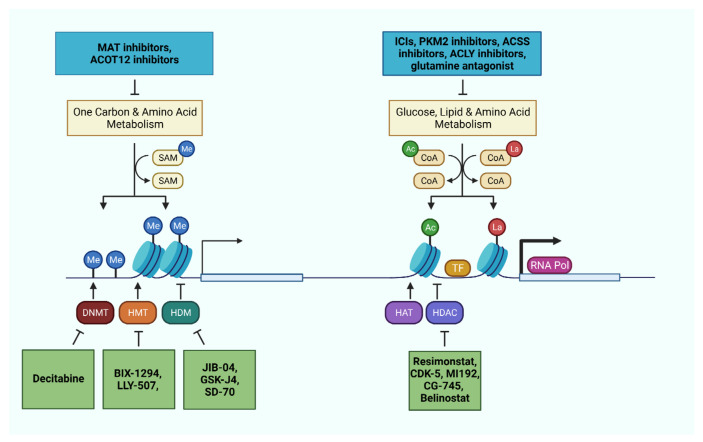
Metabolic and epigenetic machineries as therapeutic targets in liver cancer cells and immune cells. Pharmaceutical interventions targeting relevant metabolic pathways and epigenetic machineries offer the opportunity to impede disease progression, reactivate the immune system, and sensitize the tumor through combinatorial therapies.

In the last decade, many studies have been focused on the development of metabolic inhibitors as supplemental approach for cancer therapy. Targeting specific metabolic pathways might not only impact on cellular functionality but also indirectly interfere with epigenetic changes regulating genes responsible for shaping the TME (Figure 3). Here, we discuss the recent advancement of metabolic strategies for liver cancer and anti-tumor immunity (Table 2).

### 6.1. Targeting Glycolysis

The antiangiogenic and antiproliferative multikinase inhibitor sorafineb is the first line treatment for the late-stage HCC patients. Notably, the hypoxic condition induced by sustained use of sorafenib were shown to activate the NF-κB signaling, enhance glycolysis in tumor cells, and confer sorafenib-resistance in HCC [135]. Accordingly, stabilization of HIF-1α was reported to promote HCC progression and drug resistance [136].

Recent work by Martin et al. demonstrated that shikonin, a PKM2 inhibitor, can impact hepatoma cell proliferation and improve conventional chemotherapy efficiency in HCC patients [115]. In addition to PKM2, HK2 is another glycolytic enzyme overexpressed in human HCC, and the expression level positively correlated with liver disease progression (from cirrhosis to carcinoma) [137]. In murine HCC model, HK2 deficiency can ablate hepatocarcinogenesis and enhance the anti-tumorigenic effects of sorafenib [26]. However, glycolysis is also critical for T cell activation and function. For instance, targeting PKM2 to inhibit glycolysis was shown to hinder the differentiation of Th17 cells and Th1 cells [116] and the loss of HK2 impairs CD8 T cell functions as well as anti-tumor immunity [114]. Therefore, although blocking glycolysis seems to be a good strategy to inhibit the proliferation of tumor cells and sensitize tumor cells to sorafenib, unspecific pharmacologic targeting of glycolysis would also ameliorate the anti-tumor immune responses.

### 6.2. Targeting Lipid Metabolism

In addition to glycolysis, aberrant lipid metabolism is another feature of liver cancer. The suppression of ACC leads to reduced proliferation of HCC cell lines [138]. Moreover, recent evidence also indicated that the liver-specific ACC inhibitor (ND-654) alone decreases HCC incidence in a rat model, while combining ND-654 with sorafenib can further inhibit HCC incidence [124]. Lipogenesis is also critical for immune cell activation and proliferation. In 2014, Berod et al. reported that inhibiting ACC1 restrained the differentiation of Th17 cells and attenuated Th17-mediated autoimmune disease [125]. Besides, ACC1 inhibition was also reported to promote memory T cells formation [126,127]. However, the effects of ACC blockade on anti-tumor immunity are still unclear. The phase-1 clinical study of ACC inhibitor PF-05221304 in healthy subjects (NCT03597217) has been completed, whereas a phase-1b (NCT04395950) as well as a phase-2 clinical study (NCT04321031) in NASH patients are still ongoing. Given the metabolic-regulatory function of ACC inhibitor in the liver, PF-05221304 might play a therapeutic role in liver cancer, especially in the context of NASH-induced HCC, or render tumor cells more vulnerable to immunotherapy. Furthermore, ATP citrate lyase (ACLY), which catalyzes the synthesis of OAA and acetyl-CoA [139], is highly expressed in human HCC [138,140]. It was shown that targeting ACLY leads to impaired tumor cell proliferation in vitro and Akt-mediated tumorigenesis in vivo [141]. The expression of Stearoyl-CoA Desaturase 1 (SCD1) in human HCC is highly increased, and inhibition of SCD1 was shown to suppress tumor initiation and sorafenib resistance via modulating ER-stress-induced differentiation [128]. Others indicated that SCD1 could ameliorate ferroptosis of HCC cells, while activation of AMPK can downregulate SCD1, to increase ferroptosis susceptibility [129]. In addition, metformin was considered to upregulate fatty acid oxidation in the liver [142,143], and its pharmacological effect could abrogate tumor growth in pre-clinical HCC models [117,118,144]. Increasing evidence demonstrates that metformin-induced senescence in combination with sorafinib have an enhanced therapeutic effect on HCC [119,144]. Importantly, metformin was shown to have a critical effect on immune cells infiltrating liver cancer [123]. For instance, metformin can facilitate CD8^+^ T cells infiltration into the tumor, and induce the production of anti-tumoral cytokines, such as TNF-α and IFN-γ [121]. Kunisada et al. found that metformin can reduce tumor-infiltrating regulatory T cells by blocking the expression of FoxP3, which could sustain effective anti-tumor response [122]. Moreover, the expression of PD-L1 in tumor can be inhibited by using metformin, subsequently leading to enhanced anti-tumor activity of cytotoxic T cells [120]. Notably, CD147 is a transmembrane glycoprotein highly expressed on malignant cells, such as HCC [130,145]. It could increase lipogenesis (activating PI3K/AKT signaling pathway, to upregulate ACC1 and FASN) and inhibit FA oxidation (activating MAPK signaling pathway, to downregulate PPARα and CPT1A), and the increase of CD147 is associated with HCC proliferation and metastasis. In a clinical trial, anti-CD147 treatment after radiofrequency ablation had a better anti-recurrence benefit than radiotherapy alone in the CD147^+^ HCC patients [131]. Impressively, anti-CD147 CAR T cells were developed and displayed a good efficacy towards HCC in vitro and in vivo [146].

### 6.3. Targeting Glutamine Metabolism

Considering the high consumption of nutrients in TME, liver cancer makes use of extracellular glutamine to support OXPHOS and ATP supplement. Leone et al. developed a novel glutamine antagonist JHU083, which simultaneously inhibited tumor cell glycolysis and OXPHOS, but enhanced T cell OXPHOS and anti-tumor response in mouse melanoma [132]. In 2020, Jin et al. reported that glutaminase inhibitor (CB-839) alone has limited effect, but dual inhibition of glutaminase and glutamine transporter ASCT2 (by using V-9302) showed tumor inhibition in HCC models [133]. Activated T cells are also influenced by glutamine metabolism. Loss of glutaminase was reported to regulate Th17 cell differentiation and promote the function of Th1 cells and cytotoxic T cells. Inhibition of glutaminase led to increase Th1 and cytotoxic T cell number [134]. In summary, targeting glutamine metabolism can reduce liver cancer growth, and enhance anti-tumor immunity.

## 7. Epigenetic Targets in the Treatment of Liver Cancer

In the liver, mutations and altered expression of epigenetic modifiers in relation to driven metabolic rewiring are common [147], and tackling these epigenetic changes can be exploited to increase the efficacy of therapies against liver cancer [148]. Current epigenetic-regulating strategies for liver cancer therapy are reviewed here (Table 3).

### 7.1. Targeting DNA Methylation

Firstly, regulating DNA methylation, such as using the demethylation agent 5-azacytidine (5-AZA) showed significant therapeutic effects in murine models of liver cancer model [149]. A phase-II clinical trial reported that methylation inhibitor, decitabine (5-Aza-2′-deoxycytidine), resulted in beneficial clinical response and favorable toxicity profiles in patients with advanced HCC [164]. Similarly, decitabine was confirmed to have a good response in patients with liver-predominant metastases from solid tumors [150] (Figure 3). In 2018, Liu et al. reported a new DNA methylation inhibitor, Guadecitabine (SGI-110), which was shown to display an anti-proliferative role in HCC cell lines [151]. It can significantly upregulate the expression of tumor suppressor genes and even reactivate the immune system. Moreover, low dose of 5-AZA and the histone deacetylase inhibitor, entinostat, was reported to inhibit tumor cell metastasis by inhibiting the trafficking of MDSCs and promoting MDSC differentiation [152]. However, the influence of 5-AZA and decitabine on anti-tumor immunity is not well understood.

### 7.2. Targeting Histone Modifications

Given the dysregulation of histone deacetylases (HDACs) in liver cancer, HDAC inhibition was identified as a promising therapeutic approach in liver cancer. The pan-HDAC inhibitor, resimonstat, in combination with sorafenib (as a second line therapy) showed an early sign of efficiency in advanced HCC [157,158] (Figure 3). CDK-5 is another pan-HDAC inhibitor that was confirmed to dampen liver tumor growth and sorafenib-resistance [165]. Another study reported that HDAC3-specific inhibitor MI192 can significantly inhibit the growth of CCA by inducing apoptosis of cancer cells [166]. For CCA, CG200745 is another HDAC inhibitor which could exert anti-tumor function through the blockade of the Hippo pathway [167]. Moreover, HDAC inhibitor CG-745 was demonstrated to modulate the anti-tumor microenvironment promoting cytotoxic T cells and NK cells proliferation, and inhibiting regulatory T cells and MDSC function [159]. Importantly, the use of CG-745 can significantly increase the therapeutic effect of anti-PD-1 treatment in mice model. Another study demonstrated that belinostat (HDACi) can improve the anti-tumor activity combined with anti-CTLA4 therapy in an HCC model [168] (Figure 3). The phase-1/2 clinical trials for belinostat on patients with unresectable HCC showed stabilization of the disease [169], but the combined use of belinostat and immunotherapy was not evaluated in HCC patients.

In addition to inhibiting HDAC, targeting histone methyltransferase G9a was reported to limit tumor initiation through p53-induced apoptosis of transforming hepatocytes [153]. The dual inhibition of G9a and DNA methyltransferase by BIX-01294 (G9a inhibitor) and decitabine (DNMT inhibitor) showed a promising effect for HCC treatment [154]. Furthermore, blocking the enhancer of zeste homolog 2 (EZH2), a histone H3 lysine 27 methyltransferase, could increase the expression of NKG2d ligands in tumor cells, enhancing the NK cell-mediated cell killing [153]. Consistently, targeting EZH2 lead to the re-expression of the cytokine/chemokine CXCL10, which is necessary for NK cell recruitment and tumor cell eradiation [155].

Another proof-of-concept study suggested that the inhibition of chromatin reader bromodomain-4 in a murine model of NASH-induced HCC could inhibit liver disease progression and hepatocarcinogenesis [148]. The bromodomain inhibitor JQ-1 could not only enhance the host anti-tumor immunity but also regulate the expression of PD-L1 in tumor cells [162]. As one of the bromodomain-containing proteins, bromodomain and PHD finger containing-1 (BRPF1) was shown to promote the expression of oncogenes, such as E2F2 and EZH2, by facilitating the gene promoter H3K14 acetylation in liver cancer. This study also revealed that the BRPF1-specific inhibitor GSK5959 could ameliorate the tumor growth in mouse models [163].

Furthermore, the epigenetic inhibitors that target JmjC lysine demethylases (JIB-04, GSK-J4 and SD-70) were reported to reduce HCC aggressiveness [147]. Among these, the pan-JmjC inhibitor JIB-04 has a potent antitumor effect in tumor bearing mice. Therefore, modulating histone modification could be critical for liver cancer treatment.

### 7.3. Targeting Epigenetic Substrates

In addition to direct epigenetic therapy, targeting the production of metabolites which are necessary for epigenetic modulation could be a novel approach for liver cancer. The decrease of SAM or the switch of SAM producing enzyme MAT1A to MAT2A contribute to the development of hepatitis and HCC, and promote HCC tumor cell metastasis [170]. Instead exogenous administration of SAM was reported to have a therapeutic effect on NASH and NASH-induced HCC [171]. Targeting MAT2A to increase the endogenous SAM was shown to inhibit tumor cell growth in HCC models. Consistently, in HCC patients, the elevated 5-methylthionadenosine (MTA) levels positively correlated with the exhaustion of tumor infiltrated T cells, and MAT2A knockout significantly reduced T cell exhaustion [101]. Therefore, pharmacological blockade of MAT2A could result in inhibition of tumor growth and amelioration of immune surveillance (Figure 3). Besides, intracellular acetyl-CoA level is correlated with histone acetylation in HCC cells [172]. As we mentioned above, Acetyl-CoA carboxylase inhibitors can decrease HCC tumor cell lipogenesis by blocking acetyl-CoA carboxylation. Epigenetically, acetyl-CoA is a substrate for histone acetylation, so the regulation of acetyl-CoA could influence the histone modification and gene expression [173,174]. In addition, ACSS2 was reported to regulate the activity of HIF-2α by acetylation, to inhibit the HCC tumor cell metastasis [175]. Acetyl-CoA thioesterase 12 (ACOT12) is another enzyme that is critical for acetyl-CoA metabolism as well as histone acetylation. However, Lu et al. indicated that the downregulation of ACOT12 in HCC cells increased the intracellular levels of acetyl-CoA, inducing TWIST2 expression to promote tumor cell metastasis [172] (Figure 3). Moreover, the supplement of acetate and acetyl-CoA can functionally revert autophagy of tumor infiltrating T cells, and promote the production of IFN-γ through acetylation of H3K9Ac at the interferon promotor in vitro [176].

## 8. Conclusions and Outlook

To date, liver cancer remains as one of the deadliest cancer types, and its global incidence rate continues to grow steadily. The current therapeutic options for advanced liver cancer are limited and often shows unsatisfactory benefits in patients. The high degree of genomic heterogeneity contributes significantly to the challenge of developing novel therapies. Many of these driver gene mutations, in fact, govern essential cellular metabolic pathways, which are not only critical for maintaining homeostasis, but also for dictating the epigenetic plasticity. The balance between glucose metabolism, the TCA cycle, and lipid metabolism collectively maintain the level of lactate and acetyl-CoA for histone modifications, whereas the one carbon metabolism, together with amino acid metabolism, supply SAM and α-KG for DNA and histone methylation. These epigenetic alterations, in turn, often sustain or even boost cellular metabolism to facilitate disease progression. Efforts have been invested in exploiting the metabolic and epigenetic vulnerabilities of liver cancer as alternative therapeutic targets. Most of these approaches, however, showed little effect on disease progression and patient survival in previous clinical trials as single agents [169]. In view of such challenge, current research direction switched to focus on using these inhibitors in a combinatory fashion, as sensitizing agent, to yield better response. A phase I/II clinical study showed that the HDAC inhibitor, resminostat, was able to sensitize HCC to sorafenib in 90% of previously non-responsive patients [157]. However, further preclinical and clinical studies are still needed to explore the efficacy of other epigenetic modifier inhibitors that revealed promising results with a wide spectrum of action in other liver diseases.

Notably, metabolic reprogramming of cancer cells also radically reshapes the tumor immune microenvironment, through the competition of essential nutrients and the accumulation of immunosuppressive metabolites. Even though the approval of nivolumab and pembrolizumab for advanced HCC in 2020 demonstrated unprecedented superiority in therapeutic response, the majority of HCC patients is unfortunately unresponsive to this particular intervention [177]. In these cases, the immunosuppressive metabolic microenvironment as well as epigenetic perturbations are believed to be major drivers of the chronic exhaustion phenotype of immune effector cells. Hence, this dynamic triangular relationship between metabolism, epigenetics, and immune surveillance, presents as an appealing therapeutic opportunity for enhancing immunotherapy. It seems to be plausible that tampering with these pathological alterations could reinvigorate the immune system, and might improve the therapeutic outcomes of non-responders. Recently, a phase II clinical study is underway, combining anti-PD1 with either metformin or rosiglitazone to treat advanced solid tumors, including HCC, in hope of further boosting the response rate by reshaping the tumor microenvironment (NCT04114136). Another phase I clinical trial is attempting to assess the combination of anti-PD-L1 with the DNA methyltransferase inhibitor, guadecitabine, on both advanced HCC and CCA (NCT03257761).

In this direction, with the development and testing of novel metabolic and epigenetic inhibitors, it opens up new opportunities to interfere with cancer metabolism, epigenetic dysregulation, and normalize the immune microenvironment to enhance potentially anti-cancer immunity.

## Figures and Tables

**Table 1:** Metabolism-related gene mutation in liver cancer.

Gene	Mutation	Cancer Type (Frequency)	Metabolic Pathway
*TERT*	Promoter region point mutations (gain-of-function), amplifications	HCC (40%–60%) [7], CCA (~21%) [8]	Glycolyisis ↑ [9]
*TP53*	Point mutations (loss-of-function)	HCC (20%–30%) [7,10], CCA (20%–45%) [8]	Glycolyisis ↑, Oxidative phosphorylation ↑, Fatty acids synthesis ↑, Fatty acid oxidation ↓ [11]
*MYC*	Amplifications	HCC (10%–15%) [8], CCA (4%–6%) [8]	Glycolyisis ↑ [12]
*CTNNB1*	Point mutations (gain-of-function)	HCC (10%–40%) [7]	Glycolyisis ↑ [13]
*PTEN*	Point mutations (loss-of-function)	HCC (~10%) [10], CCA (~10%) [8]	Lipogenesis ↑ [14]
*KRAS*	Point mutations (gain-of-function)	HCC (5%), CCA (~25%) [8]	Lipogenesis ↑ [15]
*IDH1/2*	Point mutations (gain-of-function)	CCA (~30%) [16]	Glutaminolysis ↑, Oxidative phosphorylation ↑ [17]

↑: Increased metabolism in liver cancer compared with normal liver; ↓: Decreased metabolism in liver cancer compared with normal liver.

**Table 2:** Metabolic targets tested in vitro and/or in vivo for liver cancer and immune cells.

Targets	Drugs/Inhibitors	Function on Liver Cancer	Function on Immune Cells
HK2		Tumor cell proliferation ↓ [26] Sensitivity to sorafenib ↑ [26]	CD8 T cells activation ↓ [114] Anti-tumor immunity ↓ [114]
PKM2	Shikonin, TEPP-46	Tumor cell proliferation ↓ [115] Sensitivity to sorafenib ↑ [115]	Th1 and Th17 cell differentiation ↓ [116]
AMPK	Metformin, AICAR	Tumor cell proliferation ↓ [117,118] Sensitivity to anti-tumor drugs ↑ [119] PD-L1 expression ↓ [120] HCC tumor growth ↓ [117,118]	Tumor infiltrated CD8 T cells ↑ [121] Tumor infiltrated Tregs ↓ [122] Tumor infiltrated MDSCs ↓ [123]
ACC	Soraphen A, TOFA, ND-654, PF-05221304	Proliferation ↓ [124] Sensitivity to sorafenib ↑ [124]	Th17 cell differentiation ↓ [125] Memory T cell formation ↑ [126,127]
SCD1	SSI-4	Proliferation ↓ [128] Apoptosis ↑ [128] Ferroptosis ↑ [129] Sensitivity to sorafenib ↑ [128]	
CD147	Metuximab	Proliferation ↓ [130] Metastasis ↓ [130] Tumor recurrence ↓ [131]	
GLS	6-diazo-5-oxo-l- norleucine (DON), JHU-083, CB-389	HCC tumor growth and invasion ↓ [132,133]	Cytotoxic T cell and Th1 cell ↑ [134] Anti-tumor immunity ↑ [134]

↑: Promoted after targeted treatment. ↓: Inhibited after targeted treatment.

**Table 3:** Epigenetic targets tested in vitro and/or in vivo for liver cancer and immune cells.

Targets	Drugs/Inhibitors	Function on Liver Cancer	Function On Immune Cells
DNMTs	5-azacytidine, Decitabine, Guadecitabine (SGI-110)	Tumor growth ↓ [149,150,151] Sensitivity to sorafenib ↑ [149] Immune defense genes expression ↑ [151]	MDSCs infiltration ↓ [152]
HDMs	5-c-8HQ, GSK-J4, JIB-04, SD-70	Liver cancer progression ↓ [71]	M2 differentiation ↓ [94]
HMTs	BIX-01294, CM-272, UNC0638, JIB-04, GSK-J4, SD-70	Tumor cell apoptosis ↑ [153] Tumor cell proliferation ↓ [154] Tumor growth ↓ [153] Tumor immunogenicity (NKG2d expression) ↑ [155]	Th2 and Th17 differentiation ↑ [156] Treg and Th1 differentiation ↓ [156] NKT cell-mediated killing ↑ [153,155]
HDACs	Resimonstat, CDK-5, CG-745, MI192 (HDAC3 inhibitor), Belinostat	Tumor growth ↓ [157,158] Sensitivity to sorafenib ↑ [157,158] Anti-PD1 efficiency ↑ [159]	Cytotoxic T cells and NKT cells ↑ [159] Tregs and MDSCs ↓ [159]
HATs	B029-2 (p300 inhibitor)	Proliferation ↓ [160] Cisplatin resistance ↓ [161]	
Epigenetic Readers (e.g., BET)	JQ-1, GSK5959	PD-L1 expression ↓ [162] Oncogene (e.g., E2F2, EZH2) expression ↓ [163]	Anti-tumor immunity ↑ [162]
MAT2A		endogenous SAM ↑ [101] Tumor growth ↓ [101]	T cell exhaustion ↓ [101]

↑: Promoted after targeted treatment. ↓: Inhibited after targeted treatment.
